# Remarks by Dr. Reynell Coates

**Published:** 1842-07-30

**Authors:** 


[*Remarks.*—Perhaps the rarest circumstance connected with the foregoing case is the fact of the excessive cuticular or corneous growth being based upon the cicatrix of a burn; for such we understand to be the tenor of the letter. Cicatrices in general, and more especially those formed by burns, are usually deficient in the oily and corneous secretions of the skin, and a long time elapses before their superfices assume even the ordinary appearance of cuticle. They are liable, it is true, to a variety of tumours, many of which are extremely painful, and some of them prone to take on cancerous action. At the session of May 28th, M. Gimelle exhibited to the Academy of Medicine of Paris, a case of numerous rose-coloured, flattened, reticulated tumours, seated on the cicatrix of an incised wound, by a yataghan, in a soldier who had never laboured under syphilis. M. Velpeau was inclined to consider them as specimens of keloid, but the other academicians regarded them as simple vegetations. Much has been said of late on the cancer of cicatrices, but nearly all the masses of morbid growth, observed in such situations, have been examples of organised and vitalised tumour. When burns or mechanical abrasions are so superficial that the injury extends through the rete mucosum, but no deeper than the outer layer of the cutis vera, we have frequently observed very obstinate furfuraceous degenerations of the cuticle, which may be regarded as somewhat analogous to warts. But from whatever source the various diseases peculiar to cicatrices may have originated, and in whatever tissue they may be located, there is usually reason to presume that they are purely local in their origin, and due to the irregularity of the accidental structures in which they are seated. Those of a specific character may, indeed, be capable of propagating themselves, so as to affect the system and involve the necessity of general treatment; but there is little in the history of cutaneous diseases to induce a belief that such is the case with simple and apparently local cuticular degenerations. The scab in one form of rupia, which is sometimes compared to a horn, is not, in reality, a cuticular or corneous production, but a mere indurated morbid discharge. We may, therefore, be permitted to doubt whether the institution of any course of general alterative treatment in the case of Dr. Harris would have produced, or would now bring about a change of action in the part affected. Should the trial be made we shall feel much interest in the result.

But the extent and precise disposition of the corniferous layer of the integuments is still involved in some obscurity. That it extends to the bottom of the follicles, giving origin to the hairs and feathers of man and animals, is obvious; and these often penetrate—as extensions—far below what is ordinarily regarded as skin, into the subcutaneous cellular tissue, dipping into and forming connections with the fibres of the muscular panicle. Hence, the roots of a morbid corneous growth may lie much deeper than would be generally supposed by those who consider the cuticular formation as entirely superficial. In the common corns, the degeneration extends inwardly as well as outwardly, so that the base is frequently found far below the ordinary level of the cutis vera ; and, in the more irregular and obscure structure of the wart, we often find the corneous deposit apparently involving, but more probably merely displacing or causing the absorption of fascial and other tissues, until the tumour is found intimately connected with the periosteum, from which it might be erroneously supposed to take its rise. If, then, even in the ordinary condition of the parts, the corniferous tissue be capable of forming such deep connections, how much more singular may be the occasional effects of the disturbance of order, tissue and structure, observable in extensive cicatrices. These remarks are made, not as strictures upon the case to which they are appended, and to which they may not be directly applicable, but for the purpose of illustrating two practical surgical precepts which we consider highly important, and far too frequently neglected.

1st. In all operations by incision upon corneous malformations of a purely local character, it is necessary to carry the knife at least as deep as the superficial fascia of the part ; and in very irregular structures of this character, such as the large bleeding warts, the incisions should reach the periosteum, unless tendinous thecæ or other intangible tissues intervene.

2d. In all operations by incision upon cicatrices, we should dissect to the full depth of the structure modified by suppuration ; and whenever the step is admissable, we should include the whole cicatrix. The neglect of this precaution is a very frequent cause of mischief and want of success in operations upon even slight cases of deformity from burns.]

